# Future prevalence of type 2 diabetes in Germany: a projection until 2040 including incidence trends observed during the SARS-CoV-2 pandemic

**DOI:** 10.3389/fepid.2025.1388189

**Published:** 2025-02-18

**Authors:** T. Tönnies, D. Voeltz, S. Voß, A. Hoyer, R. Brinks

**Affiliations:** ^1^ Institute for Biometrics and Epidemiology, German Diabetes Center, Leibniz Center for Diabetes Research at the Heinrich Heine University, Düsseldorf, Germany; ^2^ Chair for Medical Biometry and Epidemiology, Faculty of Health/School of Medicine, Witten/Herdecke University, Witten, Germany; ^3^ Biostatistics and Medical Biometry, Medical School OWL, Bielefeld University, Bielefeld, Germany; ^4^ Department of Statistics, Ludwig-Maximilians-University Munich, Munich, Germany

**Keywords:** diabetes, prevalence, SARS-CoV-2, COVID-19, projection, illness-death model, mathematical model

## Abstract

**Introduction:**

Previous studies indicate that the prevalence of type 2 diabetes (T2D) will increase substantially over the coming decades. One projection from 2019 estimated an increase in prevalence in Germany by 54% to 77% (depending on future trends in incidence and mortality) between 2015 and 2040. We aim to update this projection by incorporating recently published trends in T2D incidence in Germany that include the changes during the SARS-CoV-2 pandemic.

**Materials and methods:**

We used a partial differential equation that describes the illness-death model to project the age- and sex-specific T2D prevalence among adults between 2015 and 2040. This required input data for the age- and sex-specific incidence, mortality of the general population, mortality rate ratio of people with vs. without T2D and prevalence in the initial year of the projection. We considered five scenarios with different future trends in incidence and their impact on prevalence. Using the most recently available data on T2D incidence, we assumed that the incidence remains constant as observed in 2021 for the whole projection horizon (first scenario). In further scenarios, we assumed that the observed age- and sex-specific trends in incidence between 2015 and 2021 would continue until 2025 (second scenario), 2030 (third scenario), 2035 (fourth scenario) and 2040 (fifth scenario). One additional scenario assumed that the age-specific prevalence remains constant.

**Results:**

Observed trends in incidence suggest a decrease between 2015 and 2017, and a slight upward trend thereafter until 2021 in most age groups. Depending on how long these observed increases in incidence continue, the number of people with T2D in Germany will increase from 6.8 million in 2015 to between 10.9 million and 14.2 million in 2040. These numbers correspond to increases in prevalence from 10.5% in 2015 to between 15.5% and 20.1% in 2040. In the constant prevalence scenario, the overall prevalence and number of people with T2D in 2040 was 11.4% and 8.1 million, respectively.

**Conclusions:**

The future prevalence of T2D in Germany strongly depends on how long the recently observed increasing trend in T2D incidence will continue, which warrants close monitoring of these trends in post-pandemic years.

## 1 Introduction

Previous studies indicate that the prevalence of type 2 diabetes (T2D) will increase substantially over the coming decades (1–3). For Germany, an increase in the prevalence by 54%–77% between 2015 and 2040 was projected (3). Several studies showed that the future prevalence of diabetes is strongly determined by future trends in incidence (2–4). One way to inform future trends in incidence is by extrapolating trends in incidence that were observed prior to the projection period. In this regard, one major limitation of the current projection of T2D-prevalence in Germany was the fact that observed trends in the incidence of T2D were not available at the time of analysis. Instead, the projection considered different hypothetical future trends in various scenarios, assuming the same trend for all sexes and age groups. However, more recent studies show that temporal trends in T2D-incidence differ substantially by sex and age in Germany. Prevalence projections assuming the same incidence trend across all subgroups may therefore provide an inaccurate picture of possible future scenarios against the background of currently observed trends. In addition, the COVID-19 pandemic has the potential to affect the dynamics of the T2D epidemic meaningfully, because there is strong evidence that the risk of T2D is increased after a COVID-19 infection compared to controls without COVID-19 (5–8). This should be acknowledged when projecting the future T2D prevalence, for instance by including years of the pandemic in the input data for the projection model.

To address these issues of the current projection, we aim to update the previous projection of T2D prevalence in Germany by incorporating the most recent observed trends in incidence in Germany (9). The study by Reitzle et al. (9) estimated age- and sex-specific trends in T2D-incidence in Germany between 2015 and 2021 based on data from approximately 9 million people in the statutory health insurance which corresponds to about 11% of the German population. By incorporating these new data, we overcome one main limitation of the previous projection which had to rely on hypothetical trends in incidence (3).

## 2 Materials and methods

### 2.1 Projection model

We used an illness-death model to project the age- and sex-specific prevalence of T2D between 2015 and 2040. This model describes the interplay between the prevalence, incidence, and mortality and has been used previously for prevalence projections (2–4). The model divides the population into three states: “healthy,” “ill,” and “dead”, whereby “healthy” and “ill” denote the absence and presence of T2D, respectively. Transition rates between these states are the incidence rate *i*, the mortality rate among people without T2D $m_{0}$ and the mortality rate among people with T2D $m_{1}$. All transition rates depend on the two time scales calendar time and age. The temporal change in prevalence ∂p in this model is characterized with the following partial differential equation (10):

$$\partial p=(1-p)\cdot [i-p\cdot (m_{1}-m_{0})]$$

Since $m_{0}$ is usually unknown, $p\cdot (m_{1}-m_{0})$ was substituted with the mathematically equivalent expression $\frac{\;p\cdot (MRR-1)\cdot m}{\;p\cdot (MRRR-1)+1}$, where *m* is the mortality of the general population and MRR is the mortality rate ratio $\frac{m_{1}}{m_{0}}$.

The projection of prevalence is done by numerically integrating the partial differential equation using input data for the age-specific prevalence in the initial year of the projection period (starting prevalence), the age-specific incidence, mortality of the general population and the MRR. For all analyses, we considered people aged ≥18 years. A more detailed description of the projection model for the methodologically interested reader is available in previous reports (2, 3).

### 2.2 Input data

In this study, we update a previous application of the projection model (3) by incorporating new data on trends in incidence and leaving all other input data unchanged. Details of the input data on the prevalence, mortality and the MRR can be found in Tönnies et al. (3). Briefly, the starting prevalence in the year 2010 was based on data from 70 million people in statutory health insurance, which corresponds to approximately 85% of the population in Germany (11). The mortality rate of the general population between 2010 and 2040 was based on the official population projections from the German Federal Statistical Office (12) and the MRR was based on a representative health survey (13). Since there is strong evidence of a decreasing MRR over time (14), we assumed a decrease in MRR by 2% each year between 2010 and 2040. Although the starting prevalence refers to 2010, we report results for the years 2015–2040, as it was done in the previous projection (3).

New input data for the T2D-incidence between the years 2015–2021 were based on a representative sample of approximately 9 million people in the statutory health insurance. These data are considered representative for Germany (9). Reitzle et al. (9) provide sex-specific estimates for six age-groups (<18 years, 18–34 years, 35–49 years, 50–64 years, 65–79 years, ≥80 years) for each year separately. To determine the temporal trend in incidence, we fitted a linear regression model with the natural logarithm of the incidence as the dependent variable and age, calendar year and sex as independent variables. To allow for non-linear associations, we used natural cubic splines for age with four knots and for calendar year with one knot. We also included the three-way interaction between age, calendar year and sex.

### 2.3 Projection scenarios

For all projection scenarios, we used the predicted incidence from the linear regression model described above, as the input for the years 2015–2021. For the years 2010–2014 we used the predicted incidence of 2015. For years beyond 2022, we modeled five different scenarios considering different future trends in incidence and their impact on the future prevalence. For the first scenario, we assumed that the incidence remains constant between 2021 and 2040, i.e., we used the incidence in 2021 predicted from the regression model as input for the partial differential equation for all subsequent years. In four further scenarios, we assumed that the observed age- and sex-specific trends in incidence between 2015 and 2021 will continue until 2025, 2030, 2035 and 2040, respectively, and remain constant thereafter. We obtained the future estimates for the incidence in these four scenarios by extrapolating the temporal trend in incidence from the linear regression described above. For the sixth scenario, we did not use the projection model but assumed that the sex- and age-specific prevalence in 2015 would remain constant between 2015 and 2040. To obtain the number of people with T2D, we multiplied the projected age- and sex-specific prevalence with the age- and sex-specific population size from official population projections (15). The sixth scenario (constant prevalence) is rather unlikely and not supported by empirical evidence. Nevertheless we considered it here because it is often used in other projection studies (2).

## 3 Results

**Figure 1:**
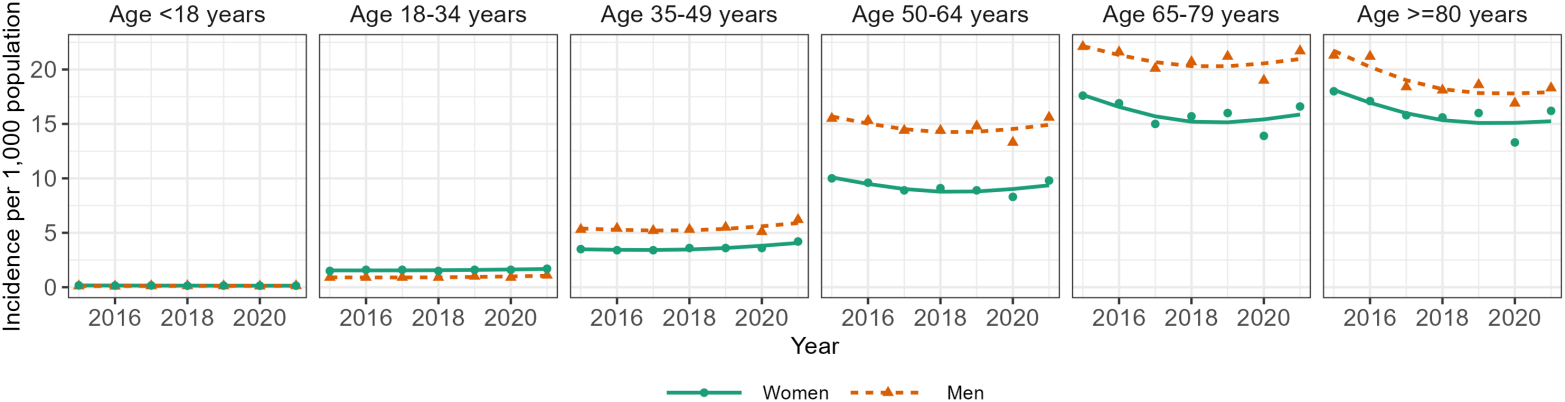
Age-specific incidence of type 2 diabetes in Germany between 2015 and 2021. Points indicate estimates as reported by Reitzle et al. (9). Lines indicate predicted values from a linear regression model with the natural logarithm of the estimates from Reitzle et al. as the dependent variable and age, calendar year, sex and their three-way interaction as independent variables.

**Figure 2:**
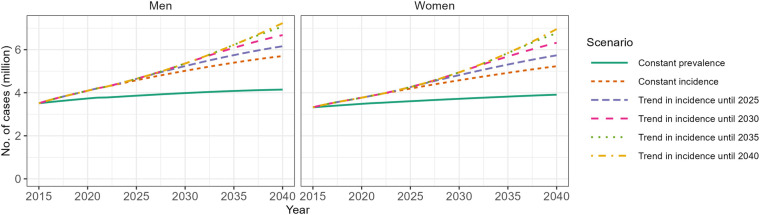
Projected number of people with type 2 diabetes (T2D) in Germany between 2015 and 2040. Scenario “Constant prevalence” assumes that the age-specific prevalence of T2D remains constant between 2015 and 2040 and only considers projected changes in the age distribution of the population. Scenario “Constant incidence” is based on the projection model that accounts for the interplay between the mortality of the general population, the mortality rate ratio of people with vs. without T2D and the incidence of T2D and assumes that the age-specific incidence of T2D remains constant between 2015 and 2040. The remaining four scenarios differ from the “Constant incidence” scenario by assuming that the observed trends in age-specific incidence (Figure 1) continue until 2025, 2030, 2035 and 2040, respectively.

**Figure 3:**
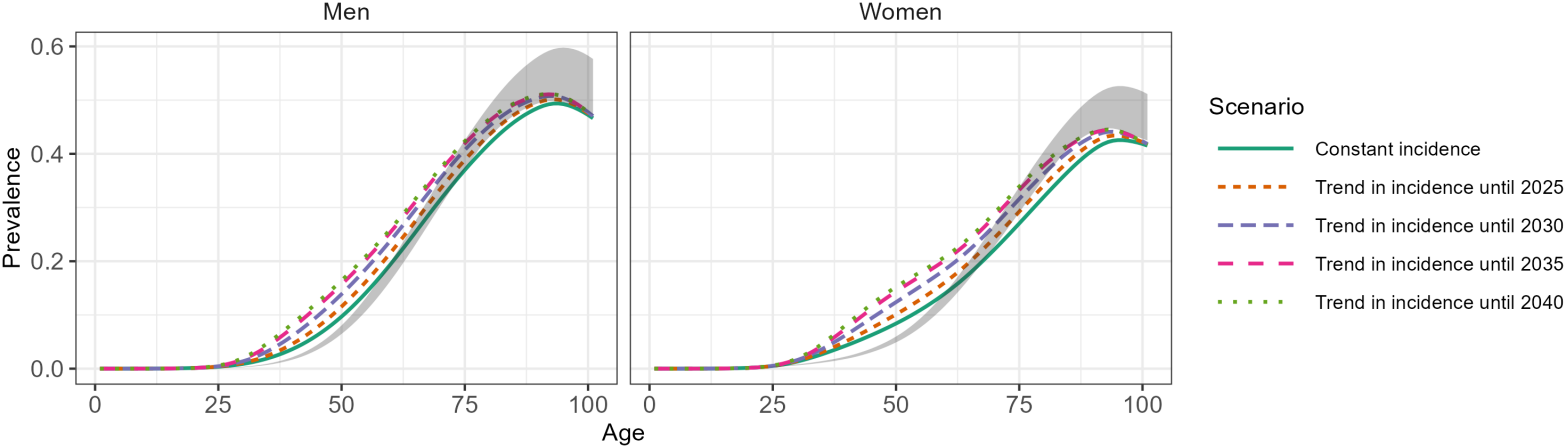
Projected age-specific prevalence of type 2 diabetes (T2D) in Germany in 2040. Scenario “Constant incidence” is based on the projection model that accounts for the interplay between the mortality of the general population, the mortality rate ratio of people with vs. without T2D and the incidence of T2D and assumes that the age-specific incidence of T2D remains constant between 2015 and 2040. The remaining four scenarios differ from the “Constant incidence” scenario by assuming that the observed trends in age-specific incidence (Figure 1) continue until 2025, 2030, 2035 and 2040, respectively. The grey-shaded areas indicates the range of the projected prevalence in 2040 from a previous projection (3).

Figure 1 shows the incidence of T2D between 2015 and 2021 by sex and age based on Reitzle et al. (9). In most age groups, the data suggest a decrease in incidence between 2015 and approximately 2017/2018, and a slight upward trend thereafter. A substantial drop in incidence is obvious for the year 2020, which is probably due to reduced health care utilization during the pandemic (16). Hence, the abrupt increase in incidence between 2020 and 2021 in some age groups may be partly driven by delayed diagnoses, which were not detected in 2020. Incorporating these incidence data into the projection model for the future number of people with T2D resulted in a substantial increase in all scenarios (Figure 2). All projection scenarios based on the illness-death model show a larger increase than the scenario assuming constant prevalence. Overall, the number of cases is projected to increase from 6.8 million in 2015 to between 10.9 and 14.2 million in the five scenarios based on the illness-death model (Table 1). In the constant prevalence scenario, the number of cases in 2040 was 8.1 million. In terms of prevalence, these numbers correspond to increases from 10.5% in 2015 to between 15.5% and 20.1% in the scenarios based on the illness-death model and to 11.4% in the constant prevalence scenario. In comparison to the previous projection (3), the current analysis resulted in substantially higher prevalences for the age group ∼30 to ∼65 years among men and women in all scenarios (Figure 3). Among women, the projected prevalences for age groups >80 years was slightly lower than in the previous projection.

**Table 1:** Projected prevalence and number of people with type 2 diabetes (T2D) in Germany between 2015 and 2040.

Sex and scenario	Prevalence in 2015 (%)	No. of cases in 2015 (million)	Prevalence in 2040 (%)	No. of cases in 2040 (million)	Relative change in prevalence (%)	Relative change in no. of cases (%)
					(2015–2040)	(2015–2040)
Men						
Constant prevalence	10.5	3.5	12.1	4.1	14	18
Constant incidence	10.5	3.5	16.6	5.7	58	62
Trend in incidence until 2025	10.5	3.5	17.9	6.2	70	75
Trend in incidence until 2030	10.5	3.5	19.4	6.7	84	90
Trend in incidence until 2035	10.5	3.5	20.6	7.1	95	101
Trend in incidence until 2040	10.5	3.5	21.1	7.2	100	106
Women						
Constant prevalence	9.5	3.3	10.8	3.9	14	18
Constant incidence	9.5	3.3	14.5	5.2	53	57
Trend in incidence until 2025	9.5	3.3	15.9	5.7	67	73
Trend in incidence until 2030	9.5	3.3	17.5	6.3	84	90
Trend in incidence until 2035	9.5	3.3	18.7	6.8	97	104
Trend in incidence until 2040	9.5	3.3	19.2	7.0	103	109
Total						
Constant prevalence	10.0	6.8	11.4	8.1	14	18
Constant incidence	10.0	6.8	15.5	10.9	55	60
Trend in incidence until 2025	10.0	6.8	16.9	11.9	69	74
Trend in incidence until 2030	10.0	6.8	18.4	13.0	84	90
Trend in incidence until 2035	10.0	6.8	19.6	13.8	96	103
Trend in incidence until 2040	10.0	6.8	20.1	14.2	101	108

Scenario “Constant prevalence” assumes that the age-specific prevalence of T2D remains constant between 2015 and 2040 and only considers projected changes in the age distribution of the population. Scenario “Constant incidence” is based on the projection model that accounts for the interplay between the mortality of the general population, the mortality rate ratio of people with vs. without T2D and the incidence of T2D and assumes that the age-specific incidence of T2D remains constant between 2015 and 2040. The remaining four scenarios differ from the “Constant incidence” scenario by assuming that the observed trends in age-specific incidence (Figure 1) continue until 2025, 2030, 2035 and 2040, respectively.

## 4 Discussion

In this study, we projected the prevalence and number of people with T2D in Germany between 2015 and 2040, incorporating recently available trends in the incidence of T2D between 2015 and 2021. This is the first projection for Germany that includes incidence trends during the SARS-CoV-2 pandemic. Depending on how long the recently observed increases in incidence continue, we found that there will be between 10.9 million and 14.2 million people with diagnosed T2D in Germany in 2040. Compared to 2015, these numbers correspond to relative increases by 55% and 101%, respectively.

Although we include trends in incidence during 2020 and 2021, it is still unclear how the SARS-CoV-2 pandemic affects current and future trends. The results from Reitzle et al. (9) show an abrupt decrease in incidence in 2020, which is likely due to decreased detection of T2D caused by fewer health care consultations during pandemic containment measures. The abrupt increase in 2021 may therefore be partly driven by delayed diagnoses of T2D. However, it also seems plausible that the pandemic indeed increased the risk of T2D on the population level, since SARS-CoV-2 infection is associated with an increased risk of T2D (5–8). Moreover, socioeconomic consequences of the pandemic and its containment measures may lead to changes in the social determinants of T2D and health-related behaviors (17). As our results show, the future burden due to T2D strongly depends on how long the currently observed trends in incidence will continue. If the trends stopped in 2021 and remain constant until 2040, we expect 10.9 million people with T2D in 2040. If the trend continues until 2040, we expect 14.2 million people with T2D. Hence, trends in incidence in the post-pandemic years should be closely monitored.

With this study, we updated a previous projection for Germany which found mostly similar numbers for 2040 with regard to the total number of people with T2D. The largest difference to our study was the maximum number of projected people with T2D. In the scenario with the largest projected prevalence, the previous study assumed an annual increase in incidence by 0.5%, which resulted in 12.3 million people with T2D in 2040. This number is considerably lower than the largest estimate in this work (14.2 million) which was based on the assumption that currently observed trends in incidence would continue until 2040. Another important difference to the previous projection refers to the age-specific prevalence. In this work, the projected prevalence in the age range from about 30–65 years of age was substantially higher than in the previous projection. This is most likely the result of increasing T2D-incidence rates observed in people younger than approximately 60 years (9, 18). The different age distribution of future T2D-prevalence in this study and the previous projection highlights the importance of using age-specific trends in incidence as input data whenever possible. At the time of the previous projection, no age-specific trends in incidence were available and the same trend was assumed for all age groups. Using age-specific trends in this study probably led to more accurate projections, particularly for age groups from (about) 30–65 years.

One strength of our study is that the projection model accounts for the dynamic interplay between the prevalence, incidence and mortality. Furthermore, we were able to incorporate recent data on trends in T2D incidence during the SARS-CoV-2 pandemic. However, a weakness of our study is that our results only refer to diagnosed diabetes and therefore underestimate the true prevalence of T2D. Moreover, our projection model did not take into account that people migrating to Germany may have higher or lower prevalence than the resident population. However, the impact of migration is probably low (10). Another limitation is the implicit assumption that medical interventions to prevent T2D will not improve. Advances in this regard could substantially alter future trends in incidence. We addressed this issue by considering multiple scenarios of future trends in incidence. Unfortunately, the projected numbers for the years 2010–2019 have not been validated yet.

In summary, our results indicate a substantial increase in the prevalence of T2D in Germany between 2015 and 2040. The amount of increase largely depends on how long the increasing trend in incidence of T2D observed in recent years will continue which warrants close monitoring of incidence trends in the post-pandemic years. This is particularly true because trends in incidence are likely influenced by changes in health care seeking behavior of the population during the pandemic years and therefore do not solely reflect true changes in incidence. Future research should also consider diseases that are closely related to T2D, such as obesity, hypertension and cardiovascular disease and that are most likely affected due to pandemic's impact on lifestyle and access to medical care.

## Data Availability

Publicly available datasets were analyzed in this study. This data can be found here: The data are available from the references cited in the methods section.
